# Unraveling gene regulation mechanisms in fish: insights into multistress responses and mitigation through iron nanoparticles

**DOI:** 10.3389/fimmu.2024.1410150

**Published:** 2024-06-14

**Authors:** Neeraj Kumar, Supriya Tukaram Thorat, Meghana Ajit Gunaware, Paritosh Kumar, Kotha Sammi Reddy

**Affiliations:** School of Edaphic Stress Management (SESM), ICAR-National Institute of Abiotic Stress Management, Baramati, India

**Keywords:** iron nanoparticles, toxicity, gene regulation, detoxification, fish

## Abstract

The recent trend of global warming poses a significant threat to ecosystems worldwide. This global climate change has also impacted the pollution levels in aquatic ecosystems, subsequently affecting human health. To address these issues, an experiment was conducted to investigate the mitigating effects of iron nanoparticles (Fe-NPs) on arsenic and ammonia toxicity as well as high temperature stress (As+NH_3_+T). Fe-NPs were biologically synthesized using fish waste and incorporated into feed formulations at 10, 15, and 20 mg kg^-1^ diet. A total of 12 treatments were designed in triplicate following a completely randomized design involving 540 fish. Fe-NPs at 15 mg kg^-1^ diet notably reduced the cortisol levels in fish exposed to multiple stressors. The gene expressions of *HSP 70*, *DNA* damage-inducible protein (*DDIP*), and DNA damage were upregulated by stressors (As+NH_3_+T) and downregulated by Fe-NPs. Apoptotic genes (*Cas 3a* and *3b*) and detoxifying genes (*CYP 450*), metallothionein (*MT*), and inducible nitric oxide synthase (*iNOS*) were downregulated by Fe-NPs at 15 mg kg^-1^ diet in fish subjected to As+NH_3_+T stress. Immune-related genes such as tumor necrosis factor (*TNFα*), immunoglobulin (*Ig*), and interleukin (*IL*) were upregulated by Fe-NPs, indicating enhanced immunity in fish under As+NH*3*+T stress. Conversely, Toll-like receptor (*TLR*) expression was notably downregulated by Fe-NPs at 15 mg kg^-1^ diet in fish under As+NH_3_+T stress. Immunological attributes such as nitro blue tetrazolium chloride, total protein, albumin, globulin, A:G ratio, and myeloperoxidase (MPO) were improved by dietary Fe-NPs at 15 mg kg^-1^ diet in fish, regardless of stressors. The antioxidant genes (*CAT*, *SOD*, and *GPx*) were also strengthened by Fe-NPs in fish. Genes associated with growth performance, such as growth hormone regulator (*GHR1* and *GHRβ*), growth hormone (*GH*), and insulin-like growth factor (*IGF 1X* and *IGF 2X*), were upregulated, enhancing fish growth under stress, while *SMT* and *MYST* were downregulated by Fe-NPs in the diet. Various growth performance indicators were improved by dietary Fe-NPs at 15 mg kg^-1^ diet. Notably, Fe-NPs also enhanced arsenic detoxification and reduced the cumulative mortality after a bacterial infection. In conclusion, this study highlights that dietary Fe-NPs can effectively mitigate arsenic and ammonia toxicity as well as high temperature stress by modulating gene expression in fish.

## 1 Introduction

The recent dramatic change in ecosystems has been witnessed to affect all living organisms, including humans, animals, and fish. Climate change, pollution, and degraded water quality are affecting the aquatic systems, resulting in the species extinction of aquatic organisms including fish (1, 2). With fish reared under a degraded environment, this results in changes at the gene and cellular levels. The contamination reaches up to cellular level of the aquatic organism, and the final product is contaminated, which increases the chances of deadly diseases occurring in consumers such as humans. Climate change and pollution can also lead to occurrences of diseases in all ecosystems. While climate change and pollution are distinct factors, they often work together to degrade the food chain and web. In aquatic systems like aquaculture and fisheries, ammonia emerges as a critical abiotic factor affecting the production and survival of aquatic animals, including fish (1). The adverse effect of abiotic factors such as arsenic (As), ammonia (NH_3_), and high temperature has weakened the immunity of aquatic organisms, which results in decreases in the efficiency of gene regulations involved in the detoxification of contamination. Moreover, arsenic pollution broadly covers the globe, including Asia, America, Europe, African countries, etc. Asian countries such as Bangladesh and the northeastern parts of India and China are also badly affected. Almost 200 million people are at a high risk (3, 4), of which 43,000 people die annually in Bangladesh due to arsenic pollution (4). As per the International Agency for Research on Cancer (IARC), it is also considered a class I carcinogenic (5). Arsenic is widely used for agriculture, veterinary drugs, medicines, metal alloy manufacturing, microelectronics, glassware, and wood preservatives (6, 7). Furthermore, the toxicity of ammonia (NH_3_) in aquatic ecosystems is crucial, resulting in mass mortality in fish. NH_3_ mainly originates from high fish protein diet, fish waste, and metabolic process of the aquatic organism, resulting in NH_3_ toxicity in aquatic systems (8, 9). The breakdown of amino acids, pyrimidines, and purines also generates ammonia (10), which exists in two forms: unionized ammonia (NH_3_) and ionized ammonium (NH_4_^+^) (11). Ammonia toxicity can lead to noticeable reductions in growth performance (12), immunity, tissue erosion, neurotoxicity, and oxidative stress and ultimately result in high mortality (13). Similarly, elevated temperature also alters the fish physiology as fish are poikilothermic animals.

Iron (Fe) is an essential nutrient which has an important role in oxygen transport and cellular respiration in fish (14). Moreover, the fish can absorb Fe using its gills and intestinal mucosa (14). In this study, multiple abiotic factors (As, NH_3_, and high temperature—T) were employed for stress which induces a weakened immunity in fish, resulting in alterations of the gene regulations involved in the immunity of fish, although immunity has been indicated and reflected from primary stress response to tertiary stress response. In the case of weak immunity, the natural kappa factor (NFkB) signaling pathway is inhibited due to multiple abiotic factor stress. Moreover, the dietary iron nanoparticles (Fe-NPs) improve the immunity of the fish using the NFkB signaling pathway (15, 16). The nano-form of iron sulfate is highly bioavailable to fish compared to other forms of iron (17). Therefore, the supplementation of Fe-NPs diet can be maximized as enhancer of immunity, anti-oxidant status, and the growth performance of the fish (18). The gene responsible for apoptosis and programmed cell death, cytokines, NF-κB pathway, immune genes, and anti-oxidant defense genes are important for regulatory mechanism (19, 20), although cytokines are vital signaling molecules released during various conditions, modulating inflammatory responses and maintaining barrier integrity (21). Therefore, Fe-NPs control the gene regulation involved in the abovementioned process. Neurotoxicity using acetylcholine esterase (AChE) was also inhibited using multiple abiotic stresses, although the dietary Fe-NPs enhanced the AChE activities (22). Fe-NPs supplementation could enhance the growth performance in the fish. It has advantage over bulk Fe as Fe-NPs have higher bioavailability and better absorption, which can promote fish growth (18). The Fe-NPs also decrease alanine amino transferase (ALT) and aspartate amino transferase (AST) and promote good fish health (18).

A mechanistic study of multiple stresses is required to understand the gene regulation involved in such process. However, *Pangasianodon hypophthalmus* is the fish species best suited to study the impact of multiple stresses and different gene regulation pathways (23, 24). *P. hypophthalmus* has high demand due to its medicinal and taste characteristics, and it has potential for diversification as an aquaculture species (25). The aims of the present study are dealt with by two major objectives, namely (1): to understand the mechanistic role of Fe-NPs in mitigating multiple stresses (abiotic and biotic) and (2) to elucidate the role of gene regulation involved in the response to multiple stress, such as a low dose of arsenic and ammonia toxicity as well as high temperature in *P. hypophthalmus*.

## 2 Materials and methods

### 2.1 Ethics statement

The institute aquaculture wet lab facilities were registered under the Committee for the Purpose of Control and Supervision of Experiments on Animals (CCSEA)—2190/GO/RReBi/SL/2022/CCSEA. The study was approved by institute PME as 7–1(PME) 2024–04. This study was in compliance with Animal Research: Reporting of *In Vivo* Experiments (ARRIVE) guidelines.

### 2.2 Experimental animals and design

The fish were taken from the National Institute of Abiotic Stress Management’s Farm Pond. The fish weighed an average of 6.02 ± 0.24 g and measured 5.12 ± 0.18 cm in size. The fish were kept in a 150-L rectangular plastic aquarium. The fish were placed in quarantine with potassium permanganate (KMnO_4_) and a 1% dip salt solution. A total of 12 treatments were designed for this experiment: iron nanoparticles supplied to the group at 10, 15, and 20 mg kg^-1^ diet with or without stressors: arsenic (As); ammonia (NH_3_); arsenic and ammonia (As+NH_3_); and arsenic, ammonia, and high temperature (As+NH_3_+T). **Table 1** presents the details of the treatments.

**Table 1:** Experimental design of the present investigation.

S. no.	Details of the treatments	Notation
1	Control	Ctr
2	Arsenic exposure and fed with control diet	As
3	Ammonia exposure and fed with control diet	NH_3_
4	Concurrent exposure to arsenic and ammonia and fed with control diet	As+NH_3_
6	Concurrent exposure to arsenic, ammonia, and high temperature and fed with control diet	As+NH_3_+T
7	Fed with iron nanoparticles at 10 mg kg^-1^ diet	Fe-NPs at 10 mg kg^-1^ diet
8	Fed with iron nanoparticles at 15 mg kg^-1^ diet	Fe-NPs at 15 mg kg^-1^ diet
9	Fed with iron nanoparticles at 20 mg kg^-1^ diet	Fe-NPs at 20 mg kg^-1^ diet
10	Concurrent exposure to arsenic, ammonia, and high temperature and fed with iron nanoparticles at 10 mg kg^-1^ diet	Fe-NPs at 10 mg kg^-1^ diet+As+NH_3_+T
11	Concurrent exposure to arsenic, ammonia, and high temperature and fed with iron nanoparticles at 10 mg kg^-1^ diet	Fe-NPs at 15 mg kg^-1^ diet+As+NH_3_+T
12	Concurrent exposure to arsenic, ammonia, and high temperature and fed with iron nanoparticles at 10 mg kg^-1^ diet	Fe-NPs at 20 mg kg^-1^ diet+As+NH_3_+T

Fe-NPs diet was fed to the fish twice a day, at 9:00 AM and 5:00 PM. Every day, the uneaten food and excrement were removed by siphoning. The APHA method (26) was used to periodically analyze the water quality parameters, and the results were recorded within the typical range for this fish’s culture (27). Every other day, the water was physically changed (2/3rd of water), and arsenic (sodium arsenite, NaAsO_2_) and (NH_4_)_2_SO_4_ were added as sources of ammonia toxicity (NH_3_) and to provide constant aeration using a compressed air pump. The As, As+NH_3_, As+NH_3_+T, and NH_3_ stressor groups were kept together by ammonium sulfate [1/10th of LC_50_ 2.0 mg L^-1^ of (NH_4_)_2_SO_4_] (12) and arsenic (1/10th of LC_50_ 2.68 mg L^-1^) (28) and high temperature (34°C) maintained with thermostatic heaters. Four experimental diets of iso-nitrogenous (35% crude protein) and iso-caloric type were prepared. Different feed ingredients were used, such as fish meal, groundnut meal, soybean meal, wheat flour, carboxymethyl cellulose (CMC), cod liver oil, lecithin, and vitamin C. The Fe-NPs free mineral mixture was prepared manually for inclusion in the diets. The heat-labile ingredients were mixed after heating the feed ingredient. In terms of proximate analysis of the diets, these were analyzed using the method of AOAC (29). Crude protein was analyzed using nitrogen content, ether extract (EE) using solvent extraction, and ash estimation using muffle furnace (550°C) (**Table 2**). Total carbohydrate % was calculated by using the following equation:

**Table 2:** Ingredient composition and proximate analysis of experimental diets (% dry matter) of iron nanoparticles (Fe-NPs) fed to *Pangasianodon hypophthalmus* for 90 days.

Feed ingredients	Fe-NPs—0 mg kg^-1^ diet	Fe-NPs—10 mg kg^-1^ diet	Fe-NPs—15 mg kg^-1^ diet	Fe-NPs—20 mg kg^-1^ diet
Soybean meal^a^	35.5	35.5	35.5	35.5
Fish meal^a^	25	25	25	25
Groundnut meal^a^	10	10	10	10
Wheat flour^a^	17.47	17.46	17.455	17.45
Sunflower oil^a^	4.5	4.5	4.5	4.5
Cod liver oil^a^	1.5	1.5	1.5	1.5
CMC^b^	2	2	2	2
Vitamin and mineral mix^c^	2	2	2	2
Vitamin C^d^	0.03	0.03	0.03	0.03
Lecithin^b^	2	2	2	2
Fe-NPs	0	0.01	0.015	0.02
Proximate composition of the diets				
Crude protein (CP)	35.22 ± 0.28	35.28 ± 0.03	35.05 ± 0.09	35.12 ± 0.03
Ether extract (EE)	9.29 ± 0.04	9.54 ± 0.12	9.57 ± 0.16	9.56 ± 0.12
Total carbohydrate (TC)	37.69 ± 0.18	37.19 ± 0.35	36.72 ± 0.45	37.45 ± 0.10
Organic matter (OM)	90.75 ± 0.04	90.19 ± 0.49	90.29 ± 0.54	90.40 ± 0.14
Dry matter (DM)	91.44 ± 0.20	91.82 ± 0.43	91.05 ± 0.18	91.73 ± 0.17
Digestible energy (DE)	356.62 ± 0.82	356.67 ± 1.21	354.05 ± 1.69	357.19 ± 1.22
Iron (Fe, mg kg^-1^)	2.62 ± 0.13	13.81 ± 0.32	19.48 ± 0.60	23.73 ± 0.97

Digestible energy (DE) (kcal/100 g) = (% CP × 4) + (% EE × 9) + (TC × 4). Data are expressed as mean ± SE; n = 3.

a Procured from the local market.

b Himedia Ltd.

c Manually prepared vitamin mineral mixture—composition of the vitamin mineral mix (quantity/250 g starch powder): vitamin A—55,00,00 IU; vitamin D3—11,00.00 IU; vitamin B1—20 mg; vitamin E—75 mg; vitamin K—1.00 mg; vitamin B12—0.6 mcg; calcium pantothenate—2.50 mg; nicotinamide—1,000 mg; pyridoxine—100 mg; Zn—500 mg; I—1.00 mg; Mn—100 mg; Cu—200 mg; Co—45 mg; Ca—50 g; P—30 g; Se—2 ppm.

d SD Fine Chemicals Ltd., India.


Total carbohydrate%=100−(CP%+ EE%+Ash %+Moisture %)

The gross energy of the diets was calculated using the method described by Halver (30).

### 2.3 Synthesis of iron nanoparticles using the green approach

#### 2.3.1 Preparation of fish tissue extract

Fish waste (gill tissue) was used to synthesize Fe-NPs. The tissues were cut open, and blood and dust were washed away with running water. After tissue homogenization (SCILOGEX D160, Serial number-DA198AB0000585; 1275 Cromwell Ave., Suite 122; Rocky Hill, CT, USA), the supernatant was recovered by centrifuging (Thermoscientific, SORVALL, Legend MICRO 21 R) at 5,000–6,000 rpm. Whatman paper (0.45-μm pore size) was then used as filter to get the gill extract (31, 32).

#### 2.3.2 Preparation and characterization of iron nanoparticles

**Figure 1:**
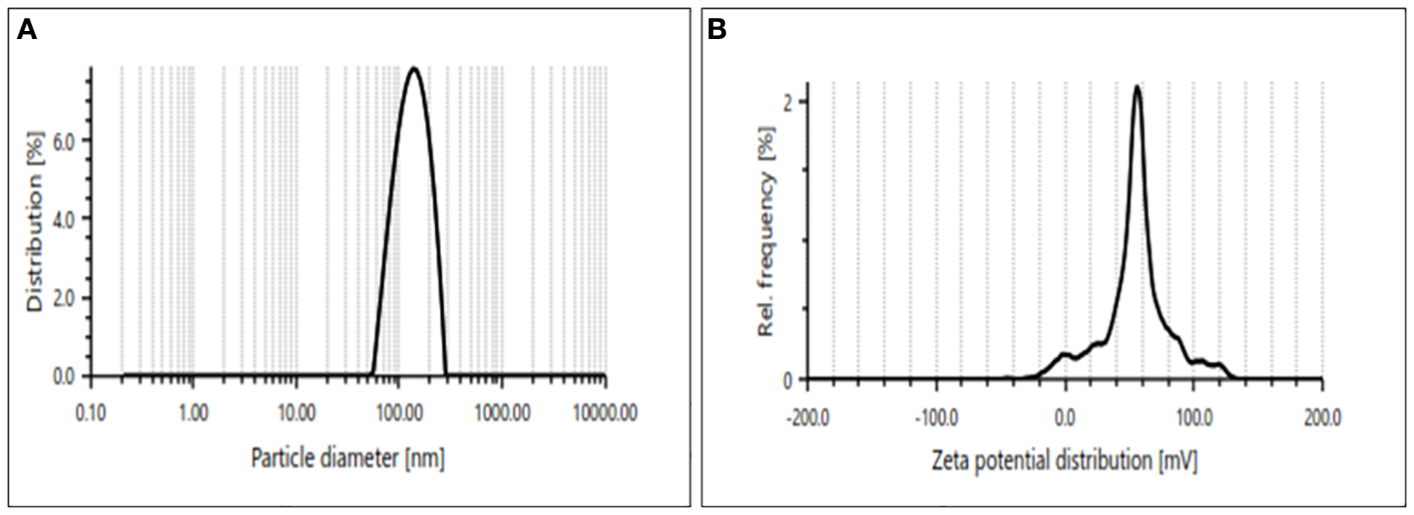
**(A, B)** Size of iron nanoparticles (Fe-NPs) 130nm and Zeta potential 53.7 mV.

Ferrous sulfate (0.4 M) was combined with the gill extract. After that, this was stirred at room temperature for 30 min. After that, the magnetic stirrer was maintained at 60 ° C for 2.5–4 h while gradually adding 0.8 M NaOH drop by drop until a reddish-brown solution is achieved. Following the acquisition of a reddish-brown hue in the solution, a spectrophotometer [UV1900i; Shimadzu (Asia Pacific) Pte Ltd., Cintech IV, Science Park I, Singapore 118264] was used to measure the peak value at (216–600 nm), where a high absorption peak was found at 250 nm. After centrifuging it and washing it three times in distilled water, pellet was obtained. The pellet was then allowed to dry in the concentrator. Iron nanoparticles (Fe-NPs) are obtained as a reddish-brown powder after drying. Additionally, the synthesized Fe-NP formulations were combined with Milli-Q water and subjected to particle size characterization (Particle Analyser, Litesizer 500, Anton Paar, Austria). The mean size and zeta potential were obtained as 130.17 nm and 53.3 mV, respectively (**Figures 1A, B**).

### 2.4 RNA isolation and quantification

Total RNA was isolated from *P. hypophthalmus* liver and muscle tissues using the TRIzol technique. The liver and muscle (*MYST* and *SMT*) tissues were homogenized using liquid nitrogen. After adding chloroform to the homogenized samples for phase separation, they were incubated for 5 min. Following centrifugation, the RNA-containing aqueous phase was separated into a 1.5-mL tube, and isopropanol was used to precipitate the RNA. After air-drying the RNA pellet, it was dissolved in RNAse-free water after the precipitated RNA had been cleaned with 75% ethanol. For later use, RNA was kept in storage at -80°C. Furthermore, 1.0% agarose gel was used to confirm the integrity of the RNA. It was made by melting agarose in 1X TAE buffer to the necessary amount. A gel documentation system (ChemiDocTM MP imaging system, Bio-Rad) was used to visualize the RNA bands. The RNA was quantified using a nano-drop spectrophotometer (Thermo-scientific).

### 2.5 cDNA synthesis and quantitative PCR

Revert Aid First Strand cDNA synthesis kit (Thermo-scientific) was used to synthesize cDNA from total extracted RNA. Prior to the creation of cDNA, trace quantities of DNA were eliminated using DNase I. A volume of 12 µL contained the reaction mixture, including 15 pmol of oligo dT primers and 100 ng of RNA. In the PCR, the reaction mixture was heated to 65°C for 5 min before being cooled on ice. With that, the chilled mixture was centrifuged for a brief period of time with the addition of 1 µL Ribo Lock RNase Inhibitor (20 U/µL), 1.0 µL of reverse transcriptase enzyme, 5X reaction buffer (4.0 µL), and 2 µL dNTP Mix (10 mM). Subsequently, the mixture was incubated for 42 min at 60°C and for 5 min at 70°C. Through the use of β-actin PCR, the generated cDNA was verified. Quantitative PCR (real-time PCR) was conducted using gene-specific primers and the SYBR green PCA master mix (Bio-Rad). SYBR Green Master Mix (1X), primer (1 µL), and 1 µL of cDNA were included in the quantification samples. The reaction cycle was set up as follows: 10 min of initial denaturation at 95°C, 39 cycles of cDNA amplification, followed by 15 s of denaturation at 95°C and 1 min of annealing at 60°C (33). **Table 3** has the primers’ specifics listed.

**Table 3:** Details of the primer for relative quantitative real-time PCR.

Gene	Primer sequence (5′ –3′)	Accession number
SOD	F-GTCCATCTTACCCGGTGCCC R-CGAGAGAAGACCCGGAACGC	XM_034299545.1
CAT	F-AGCAGGCGGAGAAGTACCCA R-GCTGCTCCACCTCAGCGAAA	XM_026919141.2
GPx	F- GTCACTGCAGGATGCAACAC R- TTGGAATTCCGCTCATTGAT	XM_026947312.2
HSP 70	F- CTCCTCCTAAACCCCGAGTC R- CCACCAGCACGTTAAACACA	XM_026934573.2
iNOS	F-ACACCACGGAGTGTGTTCGT R-GGATGCATGGGACGTTGCTG	XM_026931613.2
DNA damage inducible protein	F-CACCTTCGCCCTCGAAGTCT R-GCTCGGGTGAGGTCTCTCAG	XM_026938137.2
TNFα	F-TGGAGTTCTGCTTGCCGTGG R-GCAGCCTTTGCAGTCTCGGA	XM_026942329.2
TLR	F: TCACCACGAACGAGACTTCATCC R: GACAGCACGAAGACACAGCATC	XM_026916808.2
Ghr1	FTATTGGCTACAGCTCGCCGC R-AATCACCCCGACTGTGCTGC	XM_034306157.1
Ghrb	F-TTGAGCTTTGGGACTCGGAC R-CGTCGATCTTCTCGGTGAGG	XM_026942987.2
IGF-1X1	F-GCAACGGCACACAGACACGC R-CAGACGTTCCCTCACCATCCTCT	XM_034313382.2
IGF-1X2	F-CGAGAGCAACGGCACACAGA R-TTCTGATGGACCTCCTTACAAGATG	XM_034313383.2
IL	F- AGCAGGATCCATCAAAGTGG R- GTGCTCCAGCTCTCTGGGTA	XM_026918084.2
Ig	F- GGCCAGTAATCGTACCTCCA R- CTTCGTAAGGTCCCCACTGA	XM_026923540.2
MYST	F-GGGAAAGACCTGGCCGTGAC R-TCGAGGCCGGATTCTCGTCT	XM_026910492.2
SMT	F- CTCTGGGTGGCAGAATGAAT R- AACATGAAGAGAACGTTTTCCAG	XM_026921272.2
GH	F-CCCAGCAAGAACCTCGGCAA R-GCGGAGCCAGAGAGTCGTTC	GQ859589.1
CYP P450	F-GATTCGGCATCCGTGCGTGC R-GATGTGGCTGGGACGAGCA	NC_047599.1
MT	F-CACGGCTTTTCCTGTCCGCT R-AACAGCGCCCCCAGGTGTC	AF087935.1
Cas 3a	F-CGGCATGAACCAGCGCAAC R-TCCACCGCACCATCTGTCCC	NC_047622.1
Cas3b	F-AGCTTTCCGTGAGCTGGGCT R-TGGCTGACTTGCTGTGGTCCT	NC_047601.1
Na^+^K^+^ATPase	F-AACTACAAGCCCACGTACCA R-CTTGCCAGCCTTAAAGCCAA	XM_026923907.3
β-actin	F-CAGCAAGCAGGAGTACGATG R-TGTGTGGTGTGTGGTTGTTTTG	XM_031749543.1

SOD, superoxide dismutase; CAT, catalase; GPx, glutathione peroxidase; HSP, heat shock protein; iNOS, nitric oxide synthase; TNFα, tumor necrosis factor; TLR, Toll-like receptor; Ghr, growth hormone receptor; IL, interleukin; Ig, immunoglobulin; MYST, myostatin; SMT, somatostatin; CYP P450, cytochrome P450; MT, metallothionine; Cas 3a and 3b, caspase 3; GH, growth hormone; IGF1 and 2, insulin-like growth factor.

### 2.6 Growth performance

The study examined the growth performance parameters, including relative feed intake (RFI), weight gain (%), feed conversion ratio (FCR), specific growth rate, thermal growth coefficient (TGC), protein efficiency ratio (PER), and daily growth index (DGI). For a total of 90 days, the fish’s weight was recorded every 15 days.


FCR=Total dry feed intake(g)/Wet weight gain(g)

SGR=100(ln FBW−ln IBW)/number of days

Weight gain(%)=Final body weight (FBW)−Initial body weight (IBW)/Initial body weight (IBW)×100

Relative feed intake (FI) (%/day)=100×(TFI/IBW)

PER=Total wet weight gain (g)/crude protein intake (g)

$$\text{Thermal growth coefficient }\left(\text{TGC}\right)=({\text{FBW}}^{1/3}–{\text{IBW}}^{1/3})\times {\left(\Sigma \text{D}0\right)}^{-1},\text{ where }\Sigma \text{D}0\text{ is the thermal sum }\left(\text{feeding days}\times \text{average temperature},{}^{\circ}\text{C}\right)$$

$$\text{Daily growth index},\text{DGI }\left(\%\right)=\left({\text{FBW}}^{1/3}–{\text{IBW}}^{1/3}\right)/\text{days}\times 100$$

### 2.7 Gene study

The different genes were investigated in liver tissues in this study, viz., superoxide dismutase (*SOD*), catalase (*CAT*), glutathione-s-transferase (*GST*), heat shock protein (*HSP 70*), nitric oxide synthase (*iNOS*), cytochrome P450 (*CYP 450*), caspase 3a (*CAS 3a* and *3b*), metallothionine (*MT*), tumor necrosis factor (*TNFα*), Toll-like receptor (*TLR*), growth hormone receptor (*Ghr1* and *Ghrb*), interleukin (*IL*), immunoglobulin (*Ig*), growth hormone (*GH*), and insulin like growth factor 1 and 2 (*IGF1* and *IGF 2*), and in muscle tissue, somatostatin (*SMT*) and myostatin (*MYST*) were studied for real-time quantification.

### 2.8 Cortisol

Cortisol was determined using ELISA kit (commercially available Cortisol EIA kit, catalog no. 500360, Cayman Chemicals, USA). The assay was performed as per the instruction provided with the kit. The final reading was obtained using an ELISA plate reader (Biotek India Pvt. Ltd.).

### 2.9 Arsenic and iron analysis from fish tissues, feed, and experimental water

To measure the levels of arsenic, samples were taken from the kidney, brain, gills, liver, and muscle. On the other hand, the concentration of Fe was determined in the fish muscle and diet. Using inductively coupled plasma mass spectrometry (ICP-MS) (Agilent 7700 series, Agilent Technologies, USA) in a microwave digestion system (Microwave Reaction System, Multiwave PRO, Anton Paar GmbH, Austria, Europe), the tissues and diets were processed in accordance with the method of Kumar et al. (31, 34).

### 2.10 Alkaline single-cell gel electrophoresis/comet assay

With a slight modification (32), the alkaline single-cell gel electrophoresis/comet assay of Ali et al. (35) was used to assess DNA damage in kidney tissue. The kidney tissue (50 mg) was placed in an ice-cold homogenization buffer [20 mM EDTA; 1-X Hanks’ balanced salt solution; 10% dimethyl sulfoxide (DMSO), pH 7.0–7.5] after having been cleaned twice with chilled phosphate buffer saline (Ca^2+^- and Mg^2+^-free). The tissue was used to create a single-cell suspension, which was then suspended in phosphate saline buffer after having been centrifuged for 5 min at 4°C and 3,000 rpm to extract the cell pellets. Trypan Blue Exclusion Test was also utilized for the cell viability test (35). After coating the glass slide with 200 µL of 1% regular agarose, it was combined with 85 µL of 0.5% low melting point agarose which was added to 15 µL of cell suspension (about 20,000 cells), which was then covered with a coverslip. Furthermore, following the removal of the cover slip, 100 µL of low melting point agarose was once more applied to the slides. The slides were then left in the lysing solution (100 mM Na^2^ EDTA, 2.5 M NaCl, 10 mM Tris, pH 10, with fresh additions of 1% Triton X-100 and 10% DMSO) for an entire night at 4°C. After that, the slides were put in a horizontal gel electrophoresis unit using electrophoresis buffer (1 Mm Na^2^EDTA, 300 mM NaOH, and 0.2% DMSO, pH >13.5), and the electrophoresis unit was run for 20 min at 4°C using 15 V (0.8 V cm^-1^) and 300 mA. After that, the slides were cleaned three times using 0.4 in neutralizing buffer using Tris buffer, pH 7.5, at 0.4 M. To visualize the DNA damage, 75 µL of ethidium bromide (20 µg mL^-1^) was applied to the slides for 5 min. After that, the slides were examined under a fluorescence microscope (Leica Microsystems Ltd., DM 2000, Heerbrugg, Switzerland), and pictures were taken and examined using an Open Comet image analysis system. According to the software calculated, the metric chosen for quantifying DNA damage was percent tail DNA (i.e., % tail DNA = 100% head DNA).

### 2.11 Challenge study with *Aeromonas hydrophila*

After 90 days of feeding trial, nine fishes per replicate (total of three replicates, 27 fish per treatment) were challenged with virulent *A. hydrophila* (Hi-Media, Mumbai, India, lot no. 637–51-5 and Ref 0637 P), grown in nutrient broth for 24 h at 37°C in a BOD incubator, and harvested by centrifuging the culture broth at 10,000 rpm for 10 min at 4°C. The cells were then washed thrice in sterile PBS (pH 7.2), and the final concentration was maintained at 10^8^ CFU mL^-1^. The fishes were intraperitoneally injected with 0.15 mL of bacterial suspension in each treatment group. The fish mortality in each treatment group was recorded up to 7 days of the challenge study. The tissues were dissected out from dead fish for confirmation of *A. hydrophila* as a causative agent of death.

### 2.12 Statistics

The Statistical Package for the Social Sciences (SPSS) version 16 program has been used to process the statistical analysis of the experimental data. Using Duncan’s multiple-range tests and a one-way ANOVA (analysis of variance), the significance and treatment impact have been examined. After analysis, the data was determined to be significant at *p<*0.05.

## 3 Results

### 3.1 Cortisol

**Figure 2:**
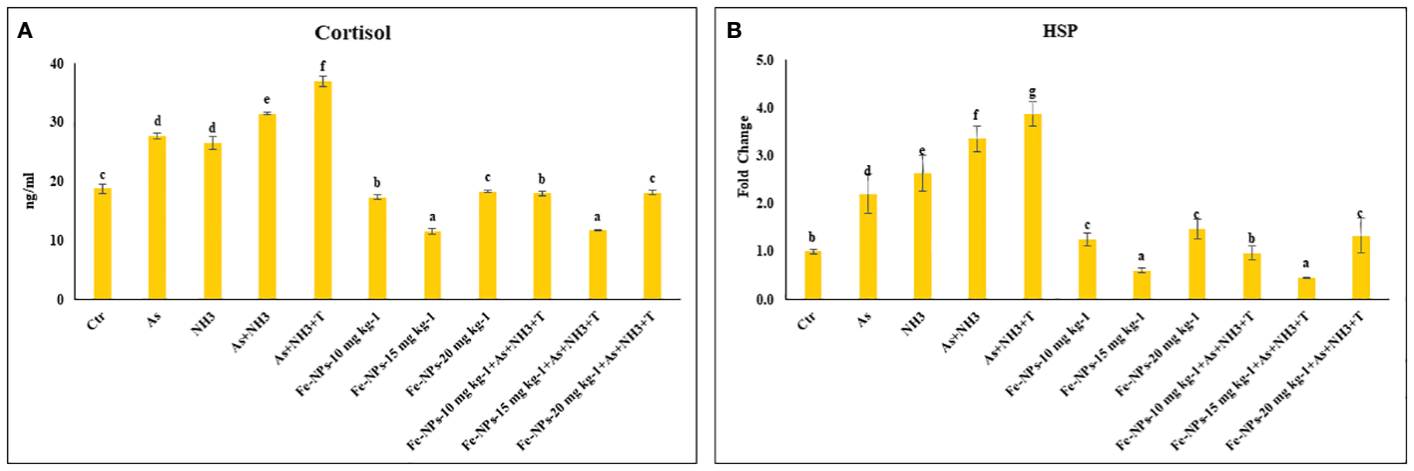
**(A, B)** Effect of dietary iron nanoparticles (Fe-NPs) on cortisol and *HSP 70* against multiple stress in fish. Within endpoints and groups, bars with different superscripts differ significantly (a-g). Data expressed as Mean ± SE (n=3).

The results of the serum cortisol levels in *P. hypophthalmus* reared under ammonia and arsenic toxicity as well as high temperature stress are illustrated in **Figure 2A**. The serum cortisol level exhibited a noticeable elevation (*p* = 0.0017) when exposed concurrently to ammonia and arsenic toxicity along with high temperature stress. This elevation was followed by concurrent exposure to ammonia and arsenic toxicity alone and then by exposure to ammonia and arsenic alone when compared to control and Fe-NP-supplemented groups. Additionally, dietary supplementation of Fe-NPs at 15 mg kg^-1^ diet, with or without stressors (As+NH_3_+T) and subsequent supplementation at 10 mg kg^-1^ diet, significantly reduced the cortisol levels compared to the control and other experimental groups.

### 3.2 Heat shock protein (*HSP 70*)

The expression of the *HSP 70* gene in liver tissue was significantly upregulated (*p* = 0.0022) by concurrent exposure to ammonia and arsenic toxicity, followed by exposure to arsenic combined with ammonia, ammonia alone, and arsenic alone, when compared to the control and other experimental groups. Interestingly, dietary supplementation of Fe-NPs at 15 mg kg^-1^ diet, with or without stressors, notably downregulated the expression of the *HSP 70* gene compared to the control and other experimental groups. Moreover, groups fed with Fe-NPs at 10 and 20 mg kg^-1^ diet, with or without stressors, showed a significantly lower expression of the *HSP 70* gene compared to all groups exposed to stressors (As, NH_3_, As+NH_3_, and As+NH_3_+T) (**Figure 2B**).

### 3.3 DNA damage inducible protein and DNA damage

**Figure 3:**
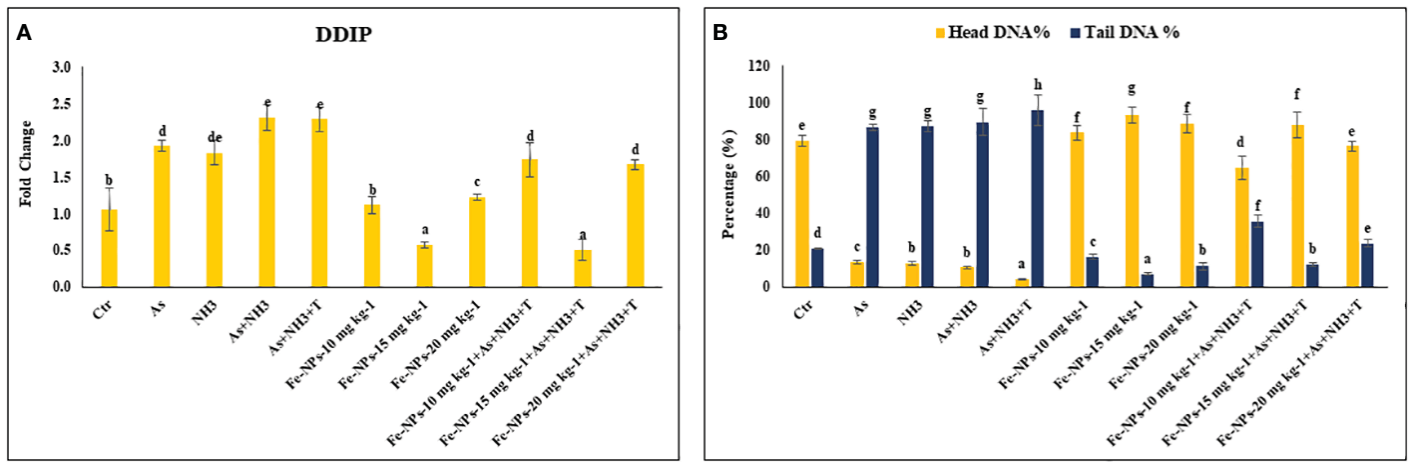
**(A, B)** Effect of dietary iron nanoparticles (Fe-NPs) on gene expression of DNA damage inducible protein (*DDIP*) and DNA damage against multiple stress in fish. Within endpoints and groups, bars with different superscripts differ significantly (a-g). Data expressed as Mean ± SE (n=3).

The gene expression of DNA damage inducible protein (*DDIP*) was notably upregulated (*p* = 0.0013) by concurrent exposure to ammonia and arsenic toxicity as well as high temperature stress, followed by exposure to arsenic combined with ammonia, ammonia alone, and arsenic alone, when compared to the control and Fe-NP-supplemented groups. Moreover, the gene expression of *DDIP* was significantly downregulated in the group fed with Fe-NPs at 15 mg kg^-1^ diet, with or without stressors (As+NH_3_+T), compared to the control and other experimental groups. Groups fed with Fe-NPs at 10 and 20 mg kg^-1^ diet, without stressors, exhibited lower gene regulation of *DDIP* compared to groups exposed to stressors (arsenic, ammonia, arsenic combined with ammonia, and arsenic combined with ammonia and high temperature) (**Figure 3A**).

Furthermore, the results of DNA damage (comet) in the form of tail DNA % and head DNA % in the gill tissue of *P. hypophthalmus* are shown in **Figure 3B**. Head DNA (%) was notably lowered (*p* = 0.016), whereas tail DNA (%) was notably higher (*p* = 0.025) in the group exposed to concurrent arsenic, ammonia, and high temperature, followed by arsenic and ammonia, ammonia alone, and arsenic alone, compared to the control and other experimental groups. The group fed with dietary Fe-NPs at 15 mg kg^-1^ diet exhibited noticeably lower tail DNA (%) and higher head DNA (%), with or without stressors, compared to the control and other experimental groups.

### 3.4 Caspase, metallothionine, and cytochrome p450

**Figure 4:**
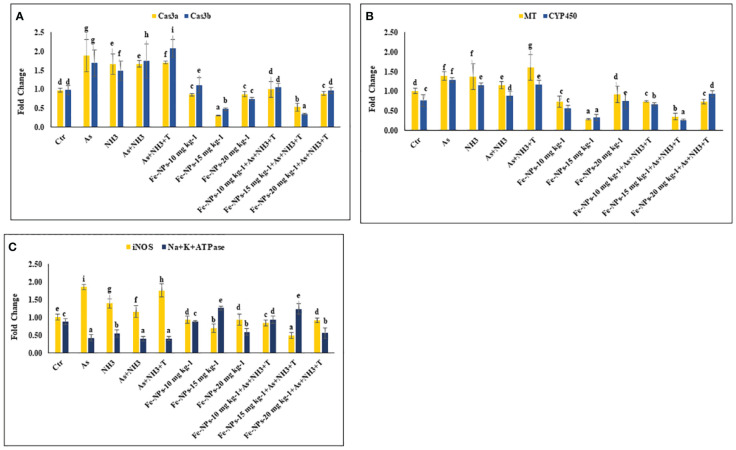
**(A–C)** Effect of dietary iron nanoparticles (Fe-NPs) on gene expression of Caspase 3a and 3b, metallothionine (*MT*), cytochrome P450 (*CYP 450*), inducible nitric oxide synthase (*iNOS*) and *Na+K+ATPase* against multiple stress in fish. Within endpoints and groups, bars with different superscripts differ significantly (a-g). Data expressed as Mean ± SE (n=3).

In the present study, the gene expression of *Cas 3a* and *3b*, *MT*, and *CYP 450*, respectively, was investigated in the liver tissue of *P. hypophthalmus* reared under multiple stress conditions (As, NH_3_, As+NH_3_, and As+NH_3_+T), and the results are presented in **Figures 4A, B**. *Cas 3a* gene expression was significantly upregulated (*p* = 0.017) by exposure to arsenic alone, followed by As+NH_3_+T, As+NH_3_, and NH_3_-alone groups. Conversely, *Cas 3b* expression was significantly upregulated (*p* = 0.0012) by concurrent exposure to arsenic, ammonia, and high temperature stress, followed by As+NH_3_, arsenic alone, and ammonia alone groups, compared to the control and other experimental groups. However, both *Cas 3a* and *3b* were downregulated by Fe-NPs at 1.5 mg kg^-1^ diet, with or without stressors, compared to the control and other experimental groups (**Figure 4A**). *MT* gene expression was substantially upregulated (*p* = 0.0031) by As+NH_3_+T, followed by arsenic, ammonia, and As+NH_3_ exposure in *P. hypophthalmus*, compared to the control and Fe-NP-supplemented groups (Fe-NPs 10 and 15 mg kg^-1^ diet). Conversely, *CYP 450* gene expression was notably (*p* = 0.016) highly upregulated by exposure to arsenic alone, followed by As+NH_3_+T, ammonia alone, and As+NH_3_ exposure, compared to the control and other experimental groups. Interestingly, *MT* and *CYP 450* gene expressions were significantly downregulated by dietary Fe-NPs at 15 mg kg^-1^ diet compared to the control and other experimental groups (**Figure 4B**).

### 3.5 Inducible nitric oxide synthase and *Na^+^K^+^ATPase*

The gene expression of inducible nitric oxide synthase (*iNOS*) and *Na^+^K^+^ATPase* in the liver tissue of *P. hypophthalmus* reared under multiple stresses is depicted in **Figure 4C**. *iNOS* gene was substantially upregulated (*p* = 0.0071) by arsenic exposure, followed by As+NH_3_+T, ammonia alone, and As+NH_3_ exposure, compared to the control and Fe-NP-supplemented groups. Notably, the *iNOS* gene was significantly downregulated with Fe-NPs at 15 mg kg^-1^ diet, with or without stressors (As+NH_3_+T), followed by Fe-NPs at 10 and 20 mg kg^-1^ diet, with or without stressors, in comparison to the control and stressor-exposed groups. Furthermore, *Na^+^K^+^ATPase* gene expression was noticeably (*p* = 0.019) highly upregulated with dietary Fe-NPs at 15 mg kg^-1^ diet, with or without stressors, compared to the control and other experimental groups. Conversely, *Na^+^K^+^ATPase* was significantly downregulated with As+NH_3_+T, As+NH_3_, arsenic-alone, and ammonia-alone exposures, compared to the control and Fe-NP-supplemented groups (**Figure 4C**).

### 3.6 Cytokines and immunological genes: tumor necrosis factor, interleukin, immunoglobulin, and toll-like receptor

**Figure 5:**
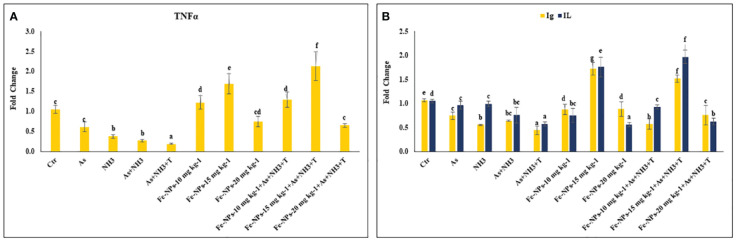
**(A, B)** Effect of dietary iron nanoparticles (Fe-NPs) on gene expression of tumor necrosis factor (*TNFa*), immunoglobin (*Ig*) and interleukin (*IL*) against multiple stress in fish. Within endpoints and groups, bars with different superscripts differ significantly (a-g). Data expressed as Mean ± SE (n=3).

**Figure 6:**
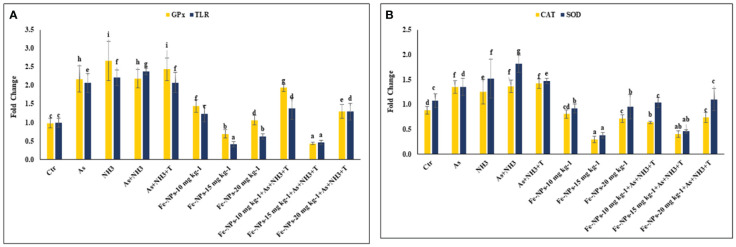
**(A, B)** Effect of dietary iron nanoparticles (Fe-NPs) on gene expression of *GPx*, toll like receptor (*TLR*), catalase (*CAT*) and superoxide dismutase (*SOD*) against multiple stress in fish. Within endpoints and groups, bars with different superscripts differ significantly (a-g). Data expressed as Mean ± SE (n=3).

The gene expression of tumor necrosis factor (*TNFα*), interleukin (*IL*), and immunoglobulin (*Ig*), respectively, were determined in the liver tissue of *P. hypophthalmus* subjected to multiple stressors, and the results are presented in **Figures 5A, B**. *TNFα* gene expression was significantly downregulated (*p* = 0.0018) by As+NH_3_+T, followed by As+NH_3_, NH_3_, and As exposure, compared to the control and other experimental groups. Conversely, *TNFα* was noticeably upregulated by dietary Fe-NPs at 15 mg kg^-1^ diet, with or without stressors, followed by Fe-NPs at 10 and 20 mg kg^-1^ diet, compared to the control and other experimental groups (**Figure 5A**). Similarly, *IL* (*p* = 0.01) and *Ig* (*p* = 0.016) gene expressions were substantially downregulated by concurrent exposure to arsenic, ammonia, and high temperature, followed by As+NH_3_, NH_3_, and As, compared to the control and diet-supplemented groups. Conversely, both *IL* and *Ig* gene expression were significantly upregulated by dietary Fe-NPs at 15 mg kg^-1^ diet, with or without stressors, compared to the control, Fe-NP-fed groups (10 and 20 mg kg^-1^ diet), and stressor-exposed groups (**Figure 5B**). Furthermore, Toll-like receptor (*TLR*) gene expression was substantially upregulated by As+NH_3_, followed by As+NH_3_+T, NH_3_, and As-alone exposure, compared to the control and other experimental groups. Moreover, Fe-NPs at 15 mg kg^-1^ diet, with or without stressors, significantly downregulated *TLR* gene expression compared to the control and stressor-exposed groups. Dietary Fe-NPs at 10 and 20 mg kg^-1^ diet supplements were less effective in mitigating multiple stressors in fish (**Figure 6A**).

### 3.7 Immunological attributes

The immunological attributes, such as nitroblue tetrazolium (NBT), blood glucose, total protein (TP), albumin (A), globulin (G), A:G ratio, and myeloperoxidase (MPO), were determined in *P. hypophthalmus* subjected to multiple stressors. The data are presented in **Table 4**. The NBT (*p* = 0.016), TP (*p* = 0.012), and globulin (*p* = 0.0022) levels were significantly decreased by concurrent exposure to arsenic, ammonia, and high temperature, followed by As+NH_3_, NH_3_, and As groups, compared to the control and Fe-NP-supplemented groups. Moreover, NBT, TP, and globulin levels were significantly elevated with dietary supplementation of Fe-NPs at 15 mg kg^-1^ diet, with or without stressors, compared to the control and other experimental groups. Furthermore, blood glucose was noticeably (*p* = 0.0027) elevated by stressors (As+NH_3_+T, As+NH_3_, NH_3_, and As) compared to the control and dietary Fe-NP-supplemented groups. Conversely, dietary Fe-NPs at 15 mg kg^-1^ diet significantly reduced the blood glucose levels in fish under both control and stressor conditions. Moreover, the A:G ratio showed similar trends to blood glucose levels, as Fe-NP supplementation improved the A:G ratio. Additionally, concurrent exposure to arsenic, ammonia, and high temperature significantly lowered the MPO levels, followed by NH_3_-alone and As-alone exposures. Interestingly, dietary Fe-NPs at 15 mg kg^-1^ diet noticeably enhanced (*p* = 0.0013) the MPO levels. The other supplemented groups of Fe-NPs at 10 and 20 mg kg^-1^ diet showed similar MPO levels to the control.

**Table 4:** Effect of dietary iron nanoparticles (Fe-NPs) on NBT, BG, total protein, albumin, globulin, A:G ratio, and MPO against multiple stress in fish.

Exposure/diets (mg kg^-1^)	NBT	BG	Total protein	Albumin	Globulin	A:G ratio	MPO
Ctr/Ctr	0.57 ± 0.03^d^	100.86 ± 3.20^c^	1.05 ± 0.07^d^	0.22 ± 0.01	0.83 ± 0.07^c^	0.27 ± 0.02^c^	0.38 ± 0.02^c^
As/Ctr	0.46 ± 0.01^c^	121.30 ± 3.11^d^	0.52 ± 0.03^c^	0.17 ± 0.02	0.36 ± 0.02^bc^	0.48 ± 0.11^d^	0.24 ± 0.01^b^
NH_3_/Ctr	0.37 ± 0.02^b^	127.19 ± 0.30^e^	0.38 ± 0.01^b^	0.13 ± 0.01	0.25 ± 0.01^b^	0.52 ± 0.07^d^	0.26 ± 0.02^b^
As+NH_3_/Ctr	0.32 ± 0.01^b^	132.35 ± 2.26^f^	0.25 ± 0.01^a^	0.12 ± 0.01	0.13 ± 0.02^a^	1.02 ± 0.30^e^	0.19 ± 0.01^a^
As+T+NH_3_/Ctr	0.21 ± 0.01^a^	148.61 ± 2.32^g^	0.24 ± 0.02^a^	0.13 ± 0.03	0.11 ± 0.01^a^	1.28 ± 0.17^f^	0.17 ± 0.01^a^
Fe-NPs-10	0.57 ± 0.03^d^	95.32 ± 2.71^b^	1.09 ± 0.02^d^	0.09 ± 0.02	1.00 ± 0.12^d^	0.09 ± 0.01^a^	0.35 ± 0.02^c^
Fe-NPs-15	0.74 ± 0.05^e^	79.83 ± 1.65^a^	1.47 ± 0.11^e^	0.15 ± 0.01	1.31 ± 0.02^e^	0.12 ± 0.01^ab^	0.47 ± 0.03^d^
Fe-NPs-20	0.56 ± 0.02^d^	103.30 ± 0.58^cd^	1.00 ± 0.04^d^	0.12 ± 0.01	0.89 ± 0.03^c^	0.13 ± 0.01^ab^	0.38 ± 0.02^c^
As+T+NH_3_/Fe-NPs-10	0.56 ± 0.01^d^	105.05 ± 0.74^d^	0.44 ± 0.03^bc^	0.11 ± 0.01	0.33 ± 0.02^bc^	0.33 ± 0.07^c^	0.32 ± 0.02^c^
As+T+NH_3_/Fe-NPs-15	0.74 ± 0.02^e^	79.45 ± 0.65^a^	1.83 ± 0.17^f^	0.19 ± 0.05	1.64 ± 0.06^f^	0.12 ± 0.03^ab^	0.46 ± 0.03^d^
As+T+NH_3_/Fe-NPs-20	0.53 ± 0.04^d^	104.23 ± 1.30^cd^	0.98 ± 0.02^d^	0.15 ± 0.02	0.83 ± 0.03^c^	0.18 ± 0.02^b^	0.38 ± 0.02^c^
*P*-value	0.016	0.0027	0.012	0.18	0.0022	0.0019	0.0013

Values in the same row with different superscript letters (a, b, c, d, e) differ significantly. Total protein, albumin, globulin: g dL^-1^ blood glucose: mg dL^-1^. Data expressed as mean ± SE (n = 3).

### 3.8 Gene related to anti-oxidative status

The gene expression of *GPx*, *SOD*, and *CAT* in the liver tissue of *P. hypophthalmus* subjected to multiple stressors is documented in **Figures 6A, B**. Exposure to As+NH_3_+T, NH_3_, As+NH_3_, and As significantly upregulated the gene expression of GPx (*p* = 0.011), SOD (*p* = 0.002), and CAT (*p* = 0.001) compared to the control and other experimental groups. Interestingly, dietary Fe-NPs at 15 mg kg^-1^ diet were effective in downregulating the gene expression of *GPx*, *SOD*, and *CAT* compared to the control and other experimental groups. The dietary supplementation of Fe-NPs at 10 and 20 mg kg^-1^ diet showed similar gene expression levels of *GPx*, *SOD*, and *CAT* as the control group.

### 3.9 Growth-related gene expression

**Figure 7:**
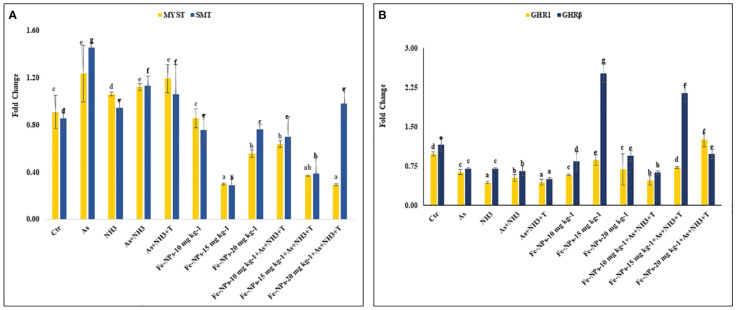
**(A, B)** Effect of dietary iron nanoparticles (Fe-NPs) on gene expression of myostatin (*MYST*), somatostatin (*SMT*), and growth hormone regulator (*GHR1* and *GHRβ*) against multiple stress in fish. Within endpoints and groups, bars with different superscripts differ significantly (a-g). Data expressed as Mean ± SE (n=3).

**Figures 7A, B** display the results of *MYST* and *SMT* gene regulation in the muscle tissue of *P. hypophthalmus* subjected to multiple stresses. *MYST* (*p* = 0.0065) and *SMT* (*p* = 0.0037) were substantially upregulated by concurrent exposure to As+NH_3_+T, followed by arsenic alone, As+NH_3_, and NH_3_ exposure, compared to the control and Fe-NP-supplemented groups. Moreover, *MYST* gene regulation was significantly downregulated by dietary Fe-NP at 15 mg kg^-1^ diet, with or without stressors, followed by Fe-NPs at 20 mg kg^-1^ diet, with or without stressors, and Fe-NPs at 10 mg kg^-1^ diet, compared to the control and other experimental groups. Similarly, *SMT* gene expression was significantly downregulated with Fe-NPs at 15 mg kg^-1^ diet, with or without stressors, followed by Fe-NPs at 10 mg kg^-1^ diet, with or without stressors, and Fe-NPs at 20 mg kg^-1^ diet without stressors, compared to the control and other experimental groups.

### 3.10 Growth hormone regulator 1 and growth hormone regulator β

*GHR1* (*p* = 0.0017) and *GHR β* (*p* = 0.0042) gene regulations were significantly downregulated in response to stressors (As+NH_3_+T, As+NH_3_, NH_3_, and As) compared to the control and other experimental groups. The *GHR1* gene expression was significantly upregulated by Fe-NPs at 20 mg kg^-1^ diet with stressors, followed by Fe-NPs at 15 mg kg^-1^ diet without stressors, compared to the control and other experimental groups. Similarly, *GHR β* was significantly upregulated by Fe-NPs at 15 mg kg^-1^ diet, with or without stressors, compared to the control and other experimental groups (**Figure 7B**).

### 3.11 Growth hormone and insulin-like growth factors (*IGF1X* and *IGF2X*)

**Figure 8:**
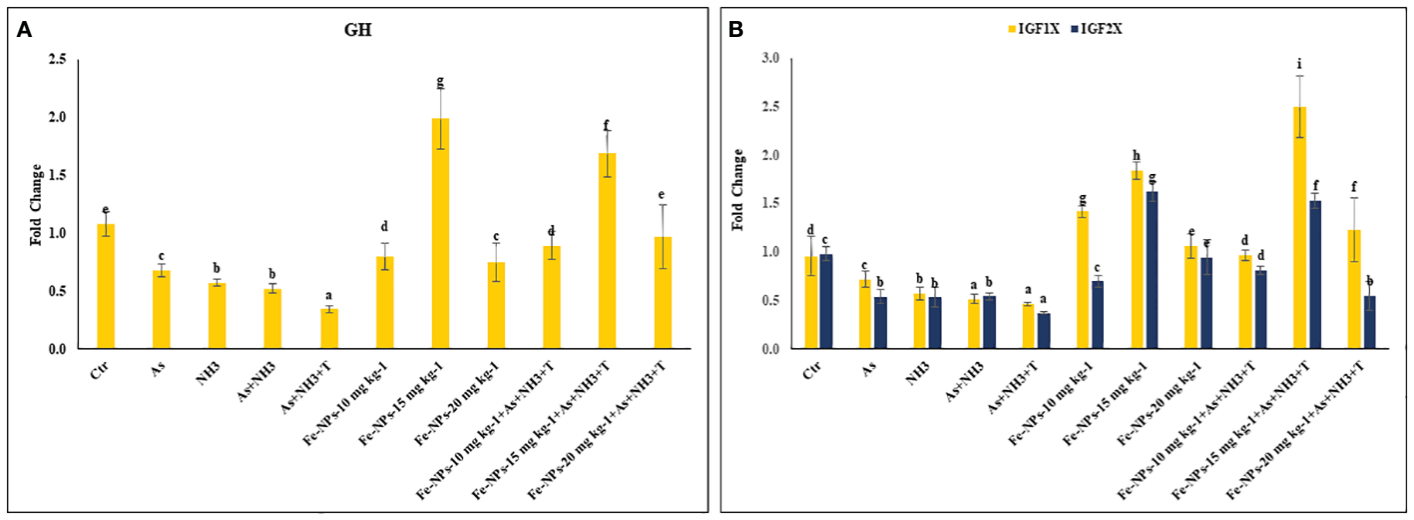
**(A, B)** Effect of dietary iron nanoparticles (Fe-NPs) on gene expression of growth hormone (*GH*), insulin like growth factor (*IGF IX* and *IGF 2X*) against multiple stress in fish. Within endpoints and groups, bars with different superscripts differ significantly (a-g). Data expressed as Mean ± SE (n=3).

The gene regulations of *GH*, *IGF1X*, and *IGF2X* in the liver tissue of *P. hypophthalmus* subjected to stressors are presented in **Figures 8A, B**. GH was substantially upregulated (*p* = 0.018) by dietary Fe-NPs at 15 mg kg^-1^ diet, with or without stressors, compared to the control and stressor-exposed groups. Conversely, *GH* gene expression was significantly downregulated by As+NH_3_+T, followed by As+NH_3_, NH_3_, and As exposures, compared to the control and supplemented-diet groups. Furthermore, *IGF1X* and *IGF2X* gene regulations were noticeably downregulated by concurrent exposure to As+NH_3_+T, followed by As+NH_3_, NH_3_, and As-alone groups, compared to the control and Fe-NP-supplemented groups. Dietary Fe-NPs at 15 mg kg^-1^ diet, with or without stressors, substantially upregulated the *IGF1X* and *IGF2X* genes compared to the control and stressor-exposed groups. Additionally, other dietary groups, such as Fe-NPs at 10 and 20 mg kg^-1^ diet, were also efficient in upregulating *IGF1X* and *IGF2X* gene regulation against arsenic, ammonia, and high temperature stress (**Figure 8B**).

### 3.12 Growth performance

**Table 5** summarizes the results of growth performance indicators such as final weight gain %, food conversion ratio (FCR), specific growth rate (SGR), protein efficiency ratio (PER), daily growth index (DGI), and relative feed intake (RFI) of *P. hypophthalmus* reared under different stress conditions. The final weight gain % was noticeably enhanced by dietary Fe-NPs at 15 mg kg^-1^ diet, with (160%) or without stressors (162%), followed by Fe-NPs with (137%) or without stressors (147%), compared to the control and stressor-exposed groups. Conversely, the final weight gain % was significantly reduced by concurrent exposure to As+NH_3_+T (60%), As+NH_3_ (68%), NH_3_ (68%), and As (72%), compared to the control and Fe-NP-supplemented groups. Similarly, SGR, PER, DGI, and RFI followed the same pattern and were enhanced with dietary Fe-NPs at 15 mg kg^-1^ diet.

**Table 5:** Effect of dietary iron nano-particles (Fe-NPs) on growth performance, viz., final body weight gain (%), FCR, SGR, PER, DGI (%), TGC, and RFI against multiple stress in fish.

Exposure/diets (mg kg^-1^)	Final body weight gain, %	FCR	SGR	PER	DGI (%)	RFI
Ctr/Ctr	96.15 ± 5.54^d^	3.15 ± 0.15^c^	0.75 ± 0.03^c^	0.91 ± 0.03^b^	1.08 ± 0.04^c^	300.92 ± 2.84^d^
As/Ctr	72.78 ± 4.70^c^	3.80 ± 0.18^d^	0.61 ± 0.02^b^	0.76 ± 0.06^a^	0.84 ± 0.02^b^	274.85 ± 4.10^c^
NH_3_/Ctr	68.02 ± 5.83^b^	3.99 ± 0.27^de^	0.58 ± 0.04^ab^	0.72 ± 0.04^a^	0.80 ± 003^b^	268.40 ± 3.52^bc^
As+NH_3_/Ctr	68.81 ± 4.33^b^	3.87 ± 0.22^de^	0.58 ± 0.03^ab^	0.76 ± 0.02^a^	0.83 ± 0.05^b^	264.32 ± 2.15^b^
As+T+NH_3_/Ctr	60.03 ± 0.97^a^	4.27 ± 0.05^e^	0.52 ± 0.01^a^	0.73 ± 0.04^a^	0.76 ± 0.04^a^	256.26 ± 1.59^a^
Fe-NPs-10	102.24 ± 2.93^e^	3.07 ± 0.07^c^	0.78 ± 0.02^c^	1.01 ± 0.01^b^	1.17 ± 0.02^d^	313.34 ± 2.55^f^
Fe-NPs-15	162.10 ± 1.88^h^	2.23 ± 0.03^b^	1.07 ± 0.01^d^	1.52 ± 0.10^e^	1.59 ± 0.02^e^	361.70 ± 1.46^i^
Fe-NPs-20	147.68 ± 9.01^g^	2.15 ± 0.15^a^	1.01 ± 0.04^d^	1.39 ± 0.04^d^	1.57 ± 0.09^e^	315.28 ± 2.77^f^
As+T+NH_3_/Fe-NPs-10	101.55 ± 7.34^e^	3.04 ± 0.18^c^	0.78 ± 0.04^c^	0.95 ± 0.08^b^	1.12 ± 0.06^d^	305.75 ± 4.21^e^
As+T+NH_3_/Fe-NPs-15	160.06 ± 0.31^h^	2.18 ± 0.01^a^	1.06 ± 0.03^d^	1.35 ± 0.03^d^	1.68 ± 0.11^f^	349.30 ± 1.85^h^
As+T+NH_3_/Fe-NPs-20	137.51 ± 6.46^f^	2.34 ± 0.09^b^	0.96 ± 0.05^d^	1.26 ± 0.07^c^	1.49 ± 0.06^e^	321.05 ± 4.32^g^
*P*-value	0.0028	0.001	0.001	0.0025	0.014	0.0011

Values in the same row with different superscript letters (a, b, c, d, e) differ significantly. Data expressed as mean ± SE (n = 3).

FCR, feed conversion ratio; SGR, specific growth rate; PER, protein efficiency ratio; DGI, daily growth index; TGC, thermal growth coefficient; RFI, relative feed intake.

### 3.13 Detoxification of arsenic by Fe-NPs diet

The arsenic concentration in the experimental water varied as follows: control (0.02 mg kg^-1^ diet), As group (1931 µg L^-1^), NH_3_ group (0.01 µg L^-1^), As+NH_3_ (2160 µg L^-1^), As+NH_3_+T (2324 µg L^-1^), Fe-NPs at 10, 15, and 20 mg kg^-1^ diet (below detection limit), and Fe-NPs with As+NH_3_+T at 10 mg kg^-1^ diet (1730 µg L^-1^), 15 mg kg^-1^ diet (507 µg L^-1^), and 20 mg kg^-1^ diet (1925 µg L^-1^). However, the results showed that dietary Fe-NPs at 15 mg kg^-1^ diet with stressors (As+NH_3_+T) exhibited the lowest arsenic concentration. Moreover, the highest bioaccumulation was observed in the kidney, followed by the liver, gill, muscle, and brain in the group exposed to As+NH_3_+T. Dietary Fe-NPs at 15 mg kg^-1^ diet showed the highest detoxification of arsenic in all tissues (**Table 6**).

**Table 6:** Effect of dietary iron nanoparticles (Fe-NPs) on detoxification of arsenic in different fish tissues reared under control and multiple stress conditions.

Exposure/diets (mg kg^-1^)	Water (µg L^-1^)	Liver (mg kg^-1^)	Kidney (mg kg^-1^)	Gill (mg kg^-1^)	Muscle (mg kg^-1^)	Brain (mg kg^-1^)	Fe-muscle (mg kg^-1^)
Ctr/Ctr	0.02 ± 0.00	BDL	0.03 ± 0.0	BDL	BDL	BDL	1.27 ± 0.03
As/Ctr	1,931.02 ± 32.88	6.65 ± 0.16	7.91 ± 0.16	3.26 ± 0.09	3.85 ± 0.23	0.52 ± 0.02	1.84 ± 0.09
NH_3_/Ctr	0.01 ± 0.0	BDL	0.14 ± 0.01	BDL	BDL	BDL	1.03 ± 0.02
As+NH_3_/Ctr	2,160.14 ± 16.22	6.84 ± 0.09	7.31 ± 0.11	2.23 ± 0.05	1.00 ± 0.02	0.83 ± 0.03	0.92 ± 0.06
As+T+NH_3_/Ctr	2,324.64 ± 25.92	8.39 ± 0.61	9.32 ± 0.02	3.49 ± 0.24	3.47 ± 0.58	0.94 ± 0.02	1.00 ± 0.16
Fe-NPs-10	BDL	BDL	BDL	BDL	BDL	BDL	4.73 ± 0.42
Fe-NPs-15	BDL	BDL	BDL	BDL	BDL	BDL	6.49 ± 0.56
Fe-NPs-20	BDL	BDL	BDL	BDL	BDL	BDL	8.95 ± 0.30
As+T+NH_3_/Fe-NPs-10	1,730.81 ± 36.43	2.96 ± 0.42	3.58 ± 0.35	2.39 ± 0.24	1.47 ± 0.16	0.86 ± 0.06	7.13 ± 0.29
As+T+NH_3_/Fe-NPs-15	507.72 ± 44.88	0.38 ± 0.08	0.47 ± 0.02	0.15 ± 0.02	0.01 ± 0.0	0.10 ± 0.01	7.32 ± 0.38
As+T+NH_3_/Fe-NPs-20	1,925.31 ± 25.91	3.99 ± 0.18	2.23 ± 0.38	1.17 ± 0.08	0.66 ± 0.06	0.80 ± 0.09	9.46 ± 0.15

Data expressed as mean ± SE (n = 3).

### 3.14 Challenging test

**Figure 9:**
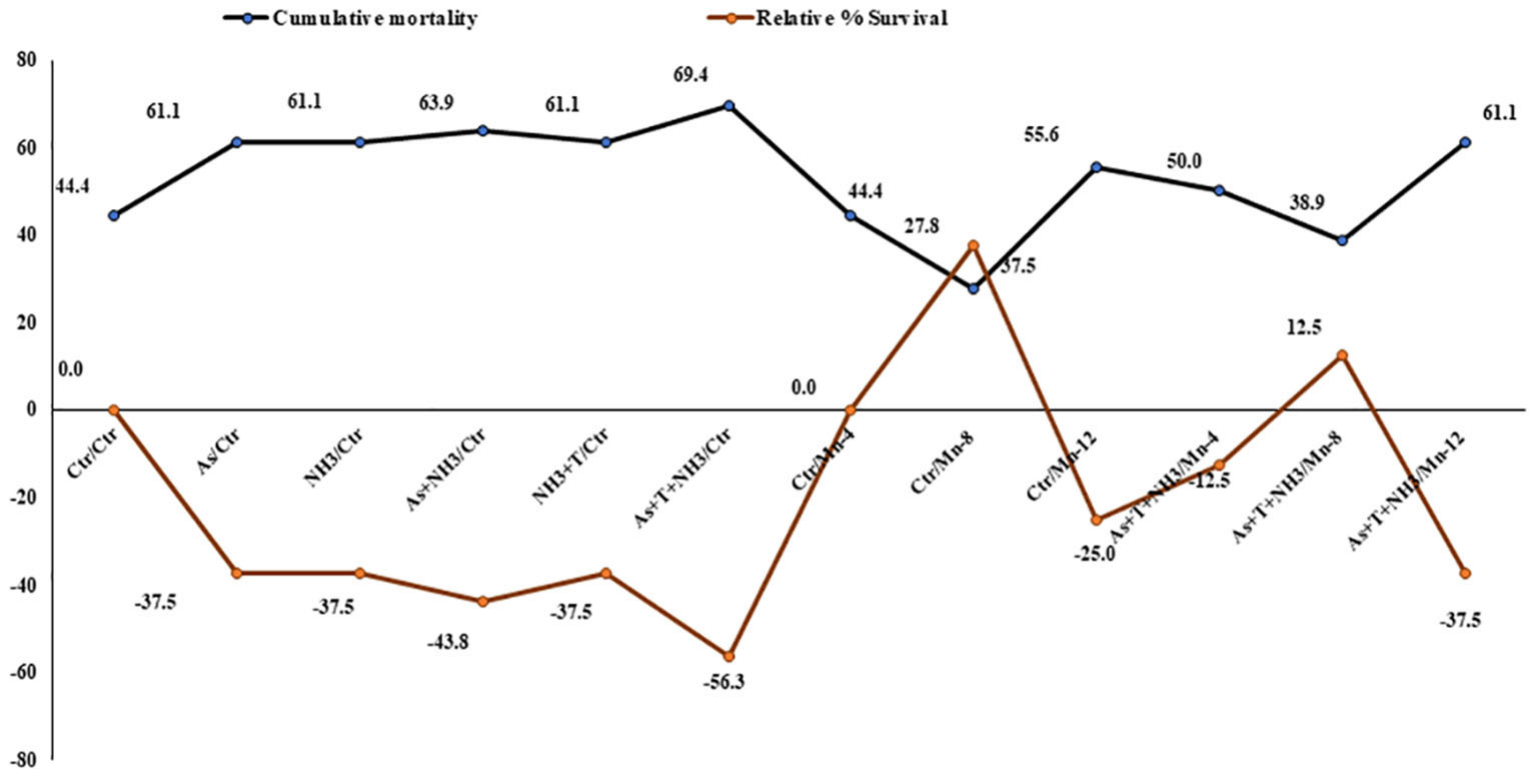
Effect of dietary iron nanoparticles (Fe-NPs) on cumulative mortality and relative percentage survival of fish reared under multiple stress after infected with pathogenic bacteria. Within endpoints and groups, bars with different superscripts differ significantly (a-g). Data expressed as Mean ± SE (n = 3).

The cumulative mortality and relative percentage survival were determined after a 90-day experimental trial. Cumulative mortality rates were observed as follows: 44%, 61%, 61%, 63.9%, 61%, 69%, 44%, 27%, 55%, 50%, and 38.9% in the control, As alone, NH_3_ alone, As+NH_3_, As+NH_3_+T, and Fe-NPs at 10, 15, and 20 mg kg^-1^ diet without stressors and with stressors, respectively. Similarly, the relative percentage survival varied as (–) 17% (–), 17% (–), 35% (–), 41%, 11%, 41%, 17%, 0%, 35%, and (–) 5.9% in the corresponding groups: control, As alone, NH_3_ alone, As+NH_3_, As+NH_3_+T, and Fe-NPs at 10, 15, and 20 mg kg^-1^ diet without stressors and with stressors, respectively (**Figure 9**).

## 4 Discussion

The present investigation highlights the critical role of dietary Fe-NPs in mitigating various stressors such as arsenic, ammonia, and high temperature (34°C) in *P. hypophthalmus*. The study primarily elucidates the mechanistic role of different gene regulations involved in immunity, antioxidative status, apoptosis, genotoxicity, growth performance, and more. It demonstrates that dietary Fe-NPs strengthen fish immunity through various gene regulations. In this study, Fe-NPs at 15 mg kg^-1^ diet substantially decreased the cortisol levels in fish reared under both control and stressor conditions. This reduction could be attributed to Fe-NPs’ activation of the hypothalamus–pituitary–interrenal (HPI) axis to control the stimulating cortisol synthesis in interrenal kidney tissue (36, 37).

Interestingly, dietary Fe-NPs reduced the cortisol levels possibly by suppressing the gene expression of *CYP 450* in fish exposed to arsenic, ammonia toxicity, and high temperature stress (38). It is also plausible that non-heme proteins containing iron, present in the adrenal cortex and involved in cortisol synthesis, play a role (39). It was also found that deficiency and overdose of iron supplementation can lead to CYP impairment. Such report has been reflected in our study (40).

The expression of the *HSP 70* gene significantly increased under stressors (As, NH_3_, As+NH_3_, and As+NH_3_+T), leading to diminished protein protection from oxidative damage, enhanced ROS production, protein misfolding, and exacerbated oxidative stress (23). Conversely, dietary Fe-NPs at 15 mg kg^-1^ diet downregulated HSP 70 gene expression, protecting against proteasomal degradation (41), promoting proper protein folding, preventing protein aggregation, degrading denatured proteins, and folding misfolded proteins (42). Surprisingly, dietary Fe-NPs downregulated the *HSP 70* gene expression, and this could be due to activation of the HSP transcription factor, which reduces the binding activity of heat shock elements (35, 43).

Furthermore, dietary Fe-NPs at 15 mg kg^-1^ diet protected against DNA damage and downregulated DDIP gene expression. Even slight changes in the dose of dietary Fe-NPs induced genotoxicity, as demonstrated in the present study, although stressors (As, NH_3_, As+NH_3_, and As+NH_3_+T) induced a higher generation of reactive oxygen species, dysregulation of cell proliferation, apoptosis, diminished DNA repair, and aberrations in histone post-translational modification and DNA methylation (44).

The gene expression of *Cas 3a* and *3b* was regulated by dietary Fe-NPs at 15 mg kg^-1^ diet, whereas stressors (As, NH_3_, As+NH_3_, and As+NH_3_+T) upregulated *Cas 3a* and *3b* (45). Iron nanoparticles (Fe-NPs) possess the protein disulfide isomerase (PDI) multifunctional enzyme systems, which control apoptosis via *Cas 3a* and *3b* (46, 47). The isomerization and rearrangement of disulfide bonds are the major functions of PDI, which has two active sites of thiol groups. It also has the capacity to bind zinc and copper at active thiol sites (48, 49).

The toxicity induced by arsenic, ammonia, and high temperature stress significantly upregulated *MT* gene expression. *MT* gene expression is commonly induced by metal toxicity and various environmental stressors in fish (50, 51). This upregulation might be mediated by the metal-responsive transcription factor 1 (MTF-1), a major activator of *MT* gene expression (52) primarily regulated by phosphorylation (53). It was reported that protein kinases such as c-Jun N-terminal kinase (JNK), phosphoinositide 3-kinase (PI3K), and protein kinase C (PKC) play roles in modifying the MTF-1 signaling pathway (53). Interestingly, dietary Fe-NPs at 15 mg kg^-1^ diet noticeably controlled and downregulated *MT* gene expression, potentially due to the essential role of iron in the erythropoietic system and the production of reticulocytes or other young cells with a basal level of MT-I much higher than that in mature erythrocytes (54).

Cytochrome P450 (*CYP 450*) is an important heme-thiolate protein involved in detoxifying xenobiotics, drugs, carcinogens, and other toxic substances (55). In this investigation, arsenic, ammonia, and high temperature stress upregulated *CYP 450* gene expression in the liver tissue of *P. hypophthalmus*. This upregulation might be attributed to reactive oxygen species generated by stressors, leading to lipid peroxidation, cellular toxicity, and, ultimately, cell death. Additionally, it is involved in apoptosis and upregulation of the transcription of bcl2-associated X (Bax) (56), an important cell death-promoting gene in fish that induces the release of cytochrome c, leading to caspase activation (57). Interestingly, dietary Fe-NPs at 15 mg kg^-1^ diet controlled the gene expression of *CYP 450* in the liver tissue of *P. hypophthalmus*.

The iNOS gene expression was significantly upregulated by stressors (As+NH_3_+T, As+NH_3_, NH_3_, and As). *iNOS* gene expression mainly depends on ammonia toxicity as it accumulates in tissues. Ammonia is converted into urea via the ornithine–urea cycle (OUC) and then into glutamine via glutamine synthetase, including non-essential amino acids (58). Similarly, blood carrying high ammonia concentrations affects the liver tissue (59). Moreover, nitric oxide provides protection to the cellular system against oxidative stress (59). Interestingly, dietary Fe-NPs at 15 mg kg^-1^ diet substantially downregulated iNOS gene expression. The interrelationship between iron and iNOS gene expression is profound, as iron nanoparticles prevent the accumulation of higher oxygen radicals through a NOS-dependent mechanism (60). Iron also triggers hypertrophy, inducing the expression of antiapoptotic proteins such as Bcl-2 and survivin and hypertrophic agents endothelin (ET-1) and leukemia inhibitory factor (LIF), thus decreasing H_2_O_2_-induced necrosis (61, 62). Furthermore, during stress conditions, the organism requires more energy in the form of ATPase; therefore, the gene expression of *Na^+^K^+^ATPase* was highly upregulated. Notably, Fe-NPs at 15 mg kg^-1^ diet aid in the formation of more ATPase and supply it to the fish reared under multiple stress conditions.

Furthermore, dietary Fe-NPs regulate the expression of cytokine genes such as *TNFα*. This regulation could be attributed to the role of iron in the regulation of ferritin levels in macrophages, enhancing the gene regulation of *TNFα* (63). In the present study, *TNFα* was substantially downregulated by stressors (As+NH_3_+T, As+NH_3_, NH_3_, and As). *TNFα* is also associated with the activation of B-cell-activating factor (BAFF), which aids in NFkB regulation (64). *TNFα* and *IL* are inflammation-related cytokines upregulated after exposure to ammonia, arsenic, and high temperature stress (65). *IL* promotes T-cell-induced inflammatory responses and releases pro-inflammatory cytokines for amplification. It binds to receptors and plays an important biological role using the nuclear factor kappa-B (NF-κB) and MAP kinase pathway (66). Additionally, TNFα is involved in apoptosis, inflammation, and cell proliferation, stimulating the immune system and activating the NF-κB pathway (67). Hence, exposure to ammonia, arsenic, and high temperature stress increases these gene regulations in fish. Moreover, dietary Fe-NPs at 15 mg kg^-1^ diet improve *IL* gene expression and upregulate its expression. This could be due to the potential of Fe-NPs to stimulate the production of hepcidin (68). The iron storage protein ferritin has two units, H-subunits (H-mRNA) and light subunits (L-mRNA), which remain unchanged, indicating that *IL* is regulated by translational mechanisms during infection and stress (69).

Furthermore, the expression of *TLR* genes was upregulated by arsenic, ammonia, and high temperature stress, whereas dietary Fe-NPs at 15 mg kg^-1^ diet downregulated *TLR* gene expression. These results could be due to heme and iron sequestration in response to inflammatory/infectious stimuli during stress (70). *TLR* also plays an important role in activating signaling pathways that enhance fish immunity and reduce stress using the NF-κB pathway. Interestingly, dietary Fe-NPs upregulated *Ig* gene expression in fish reared under control and stressor conditions (As, NH_3_, and high temperature). This strengthening of fish immunity via the upregulation of *Ig* gene expression by Fe-NPs diet could be due to enhanced immunoglobulin and T-cell levels in fish under control and stressor conditions (71, 72).

NBT, BG, total protein, albumin, globulin, A:G ratio, and MPO are also important immunological attributes whose levels indicate fish immunity. These immunological attributes were noticeably affected by exposure to arsenic, ammonia, and high temperature, leading to reduced immunity (23). This reduction could be attributed to the higher energy requirements of fish during stress conditions, resulting in significant decreases in these parameters (73). Albumin, crucial for transporting biomolecules such as drugs, hormones, vitamins, and bilirubin, also supports the regulation of hormones and fat metabolism (74). MPO produces hypochlorous acid through respiratory burst via hydrogen peroxide, exhibiting cytotoxic effects on mammals and bacteria (75). Despite higher NBT levels typically indicating better immunity, the present study observed a noticeable reduction in NBT levels under stressors (As, NH_3_, and T), reflecting compromised immunity in the fish. Surprisingly, dietary Fe-NPs at 15 mg kg^-1^ diet enhance fish immunity using these immunological attributes (71, 72). Dietary Fe-NPs help activate dendritic cells and enhance interleukin and cytokine production, accelerating the enhancement of fish immunity (76, 77).

Fe-NPs are essential nutrients for antioxidant enzymatic systems as they strengthen antioxidant gene regulation and enzymatic systems such as CAT, SOD, and GPx. In the present investigation, arsenic, ammonia, and high temperature stress substantially upregulated the gene regulation of SOD, CAT, and GPx in fish liver tissue (23). However, dietary Fe-NPs at 15 mg kg^-1^ noticeably downregulated the gene expression of SOD, CAT, and GPx, reducing reactive oxygen species (78, 79). This could be attributed to the role of Fe-NPs in maintaining oxidative stress by reducing reactive oxygen species (ROS) and acting as oxygen carrier proteins that regulate the antioxidant status of the fish (80). It is important to note that the antioxidant defense system is crucial to maintain overall health by safeguarding cells and tissues from oxidative stress damage caused by excessive hydroxyl radicals and reactive oxygen species (ROS) through scavenging them (81, 82). Moreover, Fe-NPs have many biological functions, including serving as an antioxidant defense system in fish by utilizing superoxide dismutase and glutathione peroxidase to prevent free radical production and lipid peroxidation (83). Interestingly, CAT, known as a heme-containing enzyme, acts together with SOD to counterbalance cellular ROS and improve the antioxidative status (83).

Growth performance indicators such as weight gain %, specific growth rate (SGR), protein efficiency ratio (PER), daily growth index (DGI), and relative feed intake (RFI) were noticeably reduced, and the feed conversion ratio (FCR) was enhanced with stressors (As, NH_3_, and high temperature). Conversely, dietary Fe-NPs improved the growth performance of the fish. Iron plays an important role in maintaining organismal iron homeostasis and enhances growth performance. Supplementation of Fe-NP diets enhances growth performance, possibly due to the nutrient ability of iron oxide nanoparticles and the enhancement of feed utilization in fish reared under control and stressor conditions (As, NH_3_, As+NH_3_, and As+NH_3_+T) (84). Additionally, Fe-NPs have smaller sizes and are efficiently utilized in dietary form (84). Fe-NPs diets also enhance digestive activities and increase physiological processes, which help in enhancing growth performance (85). Fe-NP diets also have higher bioavailability and better absorption, which enhances growth performance in fish reared under control and adverse conditions (86). Furthermore, genes related to growth performance such as *MYST*, *SMT*, *GHR1*, *GHRβ*, *GH*, *IGF1X*, and *IGF2X* were also altered by arsenic, ammonia, and high temperature stress. These genes were noticeably upregulated (*GHR1*, *GHRβ*, *GH*, *IGF1X*, and *IGF2X*) by dietary Fe-NPs at 15 mg kg^-1^. Dietary Fe-NPs at 15 mg kg^-1^ diet substantially downregulated MYST and SMT, possibly due to the role of *MYST* in decreasing myoblasts, resulting in terminal differentiation and fiber enlargement (87). Moreover, gene regulation of *IGF1X1* and *IGF1X2* is important for biomolecular regulation via lipid, carbohydrate, protein, and mineral metabolism and differentiation and proliferation of cells, ultimately influencing growth (18). As GH binds to the receptor in liver cells to stimulate the release and synthesis of IGF gene expression, dietary Fe-NPs aid in this process. Fe-NPs diet is responsible for growth enhancement and biomolecular function in the cells of aquatic organisms. Interestingly, Fe has a close relationship with the secretion and regulation of *IGF*, which directly affects growth performance using the central nervous system of fish reared under control and stressor conditions (88, 89). Ghrelin is also important for stimulating growth hormone from the pituitary gland, which controls the regulation of *GH*. It also stimulates the growth hormone (*GH*) releasing hormone and somatostatin axis, resulting in increased production of *IGF-I* in the liver (90).

Furthermore, the bioaccumulation of arsenic in the liver, kidney, gill, muscle, and brain was determined. The highest bioaccumulation of arsenic was observed in the kidney and liver tissues of the group concurrently exposed to arsenic, ammonia, and high temperature stress. Interestingly, in the group fed with Fe-NPs at 15 mg kg^-1^ diet with stressors, the arsenic levels were notably reduced. Arsenic levels below the detection limit were observed in the groups fed with Fe-NPs at 10, 15, and 20 mg kg^-1^ diet without stressors. These results could be attributed to the role of Fe-NPs in enhancing the detoxification of arsenic in kidney and liver tissues through *CYP 450* gene expression, as shown in the results.

Moreover, the survival rate of the fish was higher in the groups fed with Fe-NPs at 10, 15, and 20 mg kg^-1^ diet with or without stressors after bacterial challenge. Dietary Fe-NPs possess strong antibacterial and phagocytic capacities, which enhance the immunity of the fish and reduce mortality after bacterial infection. Moreover, in the present study, the immunity-related genes have been improved by dietary Fe-NPs diet. Genes such as TNFα, TLR, Ig, and IL strongly support the immunity during stress conditions, especially during infection condition (12). Fe-NPs also enhance the fluidity of membranes of antibacterial compounds like lysozyme and reduce lipid peroxidation (91–93).

## 5 Conclusions

The present investigation revealed that nano-nutrients in the form of iron nanoparticles (Fe-NPs) mitigate multiple abiotic stresses such as ammonia and arsenic toxicity as well as high temperature stress in fish. Moreover, it is the first to report the mechanistic role of Fe-NPs in mitigating arsenic and ammonia toxicity as well as high temperature stress in fish. Fe-NPs regulate genes involved in growth performance, immunity, antioxidative capacity, genotoxicity, and stress responses in *P. hypophthalmus*. Additionally, Fe-NPs lead to the positive modulation of gene expression related to cortisol regulation, *HSP 70* production, apoptosis inhibition, and genotoxicity prevention. Interestingly, Fe-NPs at 15 mg kg^-1^ diet enhance the detoxification of arsenic in fish tissues. Fe-NPs have also improved immunity against pathogenic bacteria and enhanced fish survival under multiple stresses. Importantly, these findings highlight the potential of Fe-NPs at 15 mg kg^-1^ in enhancing the wellbeing of fish species in the face of contemporary challenges posed by climate change and pollution.

## Data availability statement

The datasets for this article are not publicly available due to concerns regarding participant/patient anonymity. Requests to access the datasets should be directed to the corresponding author.

## Ethics statement

The animal study was approved by Prioritization, Monitoring and Evaluation (PME). The study was conducted in accordance with the local legislation and institutional requirements.

## Author contributions

NK: Writing – review & editing, Writing – original draft, Visualization, Validation, Supervision, Software, Resources, Project administration, Methodology, Investigation, Funding acquisition, Formal analysis, Data curation, Conceptualization. ST: Writing – review & editing, Methodology, Formal analysis. MG: Writing – review & editing, Methodology. PK: Writing – review & editing, Validation, Methodology, Data curation. KR: Writing – review & editing, Supervision.
